# Combining 4D Flow MRI and Complex Networks Theory to Characterize the Hemodynamic Heterogeneity in Dilated and Non-dilated Human Ascending Aortas

**DOI:** 10.1007/s10439-021-02798-9

**Published:** 2021-06-02

**Authors:** Karol Calò, Diego Gallo, Andrea Guala, Jose Rodriguez Palomares, Stefania Scarsoglio, Luca Ridolfi, Umberto Morbiducci

**Affiliations:** 1 PolitoBIOMed Lab, Department of Mechanical and Aerospace Engineering, Politecnico di Torino, Corso Duca degli Abruzzi 24, 10129 Turin, Italy; 2 Vall d’Hebron Institut de Recerca (VHIR), Barcelona, Spain; 3 CIBER-CV, Instituto de Salud Carlos III (ISCIII), Madrid, Spain; 4 Department of Cardiology, Hospital Universitari Vall d’Hebron, Barcelona, Spain

**Keywords:** Ascending aorta aneurysm, Aortic dilation, Magnetic resonance imaging, Network science, Spatiotemporal analysis

## Abstract

Motivated by the evidence that the onset and progression of the aneurysm of the ascending aorta (AAo) is intertwined with an adverse hemodynamic environment, the present study characterized *in vivo* the hemodynamic spatiotemporal complexity and organization in human aortas, with and without dilated AAo, exploring the relations with clinically relevant hemodynamic and geometric parameters. The Complex Networks (CNs) theory was applied for the first time to 4D flow magnetic resonance imaging (MRI) velocity data of ten patients, five of them presenting with AAo dilation. The time-histories along the cardiac cycle of velocity-based quantities were used to build correlation-based CNs. The CNs approach succeeded in capturing large-scale coherent flow features, delimiting flow separation and recirculation regions. CNs metrics highlighted that an increasing AAo dilation (expressed in terms of the ratio between the maximum AAo and aortic root diameter) disrupts the correlation in forward flow reducing the correlation persistence length, while preserving the spatiotemporal homogeneity of secondary flows. The application of CNs to *in vivo* 4D MRI data holds promise for a mechanistic understanding of the spatiotemporal complexity and organization of aortic flows, opening possibilities for the integration of *in vivo* quantitative hemodynamic information into risk stratification and classification criteria.

**Supplementary Information:**

The online version of this article (10.1007/s10439-021-02798-9).

## Introduction

Ascending aorta (AAo) aneurysm is a dilation of the segment of the aorta proximal to the brachiocephalic trunk. To avoid life-threatening complications such as dissection and rupture, AAo aneurysms are repaired by elective surgery, recommended mainly on the basis of the maximum diameter (with a fixed threshold of 5.5 cm in the vast majority of cases).^10^,^38^ However, surgical repair is associated with significant mortality rates (3–5%) 27 and aortic diameter alone has proved to be an ineffective predictor of events.^15^,^38^ These aspects have motivated research on mechanisms behind AAo aneurysm evolution as well as on new criteria for risk stratification. Although AAo dilation is the result of a multifactorial process involving genetics expressions, biological and structural factors,^15^,^48^ there is evidence of the role played by local adverse hemodynamics in flow-mediated mechanisms leading to adverse vascular remodeling.^24^,^39^ In particular, hemodynamics in dilated AAo is characterized by abnormal blood flow that reflects in near-wall flow disturbances^3^,^6^,^43^,^44^ leading to mechanical alterations in the aortic wall.^6^,^11^,^13^,^23^,^26^ Furthermore, hemodynamic disturbances are intertwined with and exacerbated by concomitant AAo dilation and aortic valve abnormalities, such as bicuspid aortic valve (BAV) or deficient tricuspid aortic valves (TAV).^14^,^28^,^36^,^46^

In recent years, 4D flow magnetic resonance imaging (MRI) has been increasingly used to obtain information on both aortic morphology and hemodynamics 5,14,31,35,43—in particular in the presence of aortic vascular/valve pathologies^4^,^16^,^23^,^31^—providing risk markers of AAo wall degeneration.^14^,^25^,^43^

With the objective of providing a comprehensive characterization of the spatiotemporal heterogeneity of large-scale aortic flow features and of their possible links with AAo dilation, a recently proposed approach integrating computational hemodynamics with Complex Networks (CNs) theory^7^,^8^,^13^,^37^ is here extended for the first time to 4D flow MRI in patients with and without aortic dilation. In this study we explored the capability of CNs to characterize *in vivo* the dynamics of dominant aortic flow features using the time-histories of the measured velocity data along the cardiac cycle. Patient-specific CNs were built with MRI voxels belonging to the aortic fluid domain as *nodes* of the networks, which were connected by *links* based on the strength of the pairwise correlation between velocity time-histories. CNs metrics allowed for the evaluation of the anatomical and topological length of correlation persistence of velocity time-histories within the aorta and its association with clinically relevant hemodynamic and geometric parameters. In prospect, the application of CNs can lead to a deeper understanding of the factors and basic mechanisms influencing the spatiotemporal complexity of aortic flows. The improved characterization of the disease has the potential to strengthen the clinical utility of blood flow visualizations, enhance diagnostic strategies and tools in terms of possible application to risk stratification and classification criteria, and inform clinical decision-making.

## Materials and Methods

Ten patients were enrolled for this study, five of them presenting with AAo dilation (one of them with bicuspid aortic valve, BAV), and five without AAo dilation (one with BAV).^14^ Ascending aortic dilation was diagnosed according to the protocol proposed elsewhere.^9^ Briefly, ascending aortic dilation was defined as at least 1.96 standard deviations above the normal diameter for a specific patient either at the aortic root or at the AAo, at the level of the pulmonary artery bifurcation, using previously proposed reference values.^9^ All patients presented with mild to severe aortic valve dysfunction (Table 1). The study was approved by the ethics committee of the Vall d’Hebron Hospital and informed consent was obtained from all participants.

**Table 1:** Aortic dilation and valve type and functional classification of the included patients.

	Patient	TAV/BAV	Aortic valve insufficiency	Aortic valve stenosis
Non-dilated AAo	A	BAV	Severe	Absent
	B	TAV	Moderate	Severe
	C	TAV	Absent	Moderate
	D	TAV	Severe	Absent
	E	TAV	Mild	Severe
Dilated AAo	F	TAV	Mild	Absent
	G	TAV	Mild	Absent
	H	BAV	Mild	Severe
	J	TAV	Mild	Absent
	K	TAV	Severe	Absent

## 4D flow MRI Acquisition and Data Processing

All patients underwent 4D flow MRI acquisitions of the entire thoracic aorta with retrospective ECG gating during free-breathing and no endovenous contrast agent. Briefly, a radially undersampled acquisition with five-point balanced velocity encoding^29^ was used. Phase-contrast MRI acquisitions were set according to the following scheme: velocity encoding in the range of 150–400 cm s^−1^, field of view 400 × 400 × 400 mm, scan matrix 160 × 160 × 160, voxel size 2.5 × 2.5 × 2.5 mm, and number of cardiac frames in the range of 30–46. Full details on the adopted 4D flow MRI acquisition protocol are reported in previous studies.^14^,^29^

**Figure 1:**
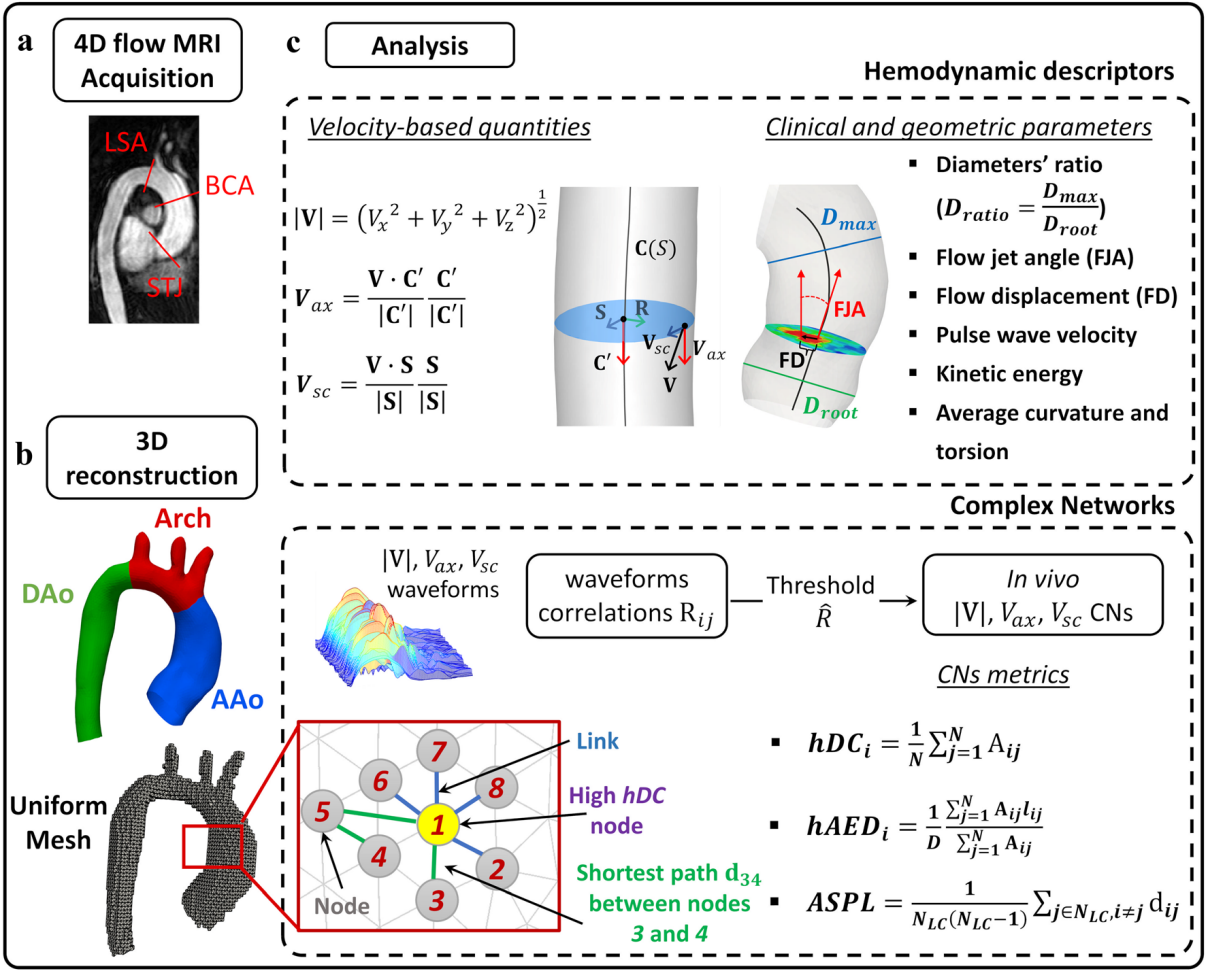
Schematic diagram of the methodology used to apply the Complex Networks theory to *in vivo* velocity data. **a**: Image acquisition and anatomic landmarks identification from 4D flow MRI: STJ: sinotubular junction; BCA brachiocephalic artery; LSA left subclavian artery. **b**: Lumen 3D geometry reconstruction. C: Definition of hemodynamic descriptors and complex networks analysis. Hemodynamic descriptors panel: *V*_*x*_, *V*_*y*_ and *V*_*z*_: cartesian components of the velocity vector **V**; **C**(*S*): vessel centerline; **C’**: vector tangent to the centerline; **R**: vector orthogonal to C’ directed from the centerline to a generic voxel; **S**: vector orthogonal to vectors **R** and **C’**. The investigated clinical and geometric parameters are also listed, with a schematic representation of flow jet angle (FJA), flow displacement (FD), the AAo maximum diameter *D*_max_ and the aortic root maximum diameter *D*_root_, the latter used to compute *D*_ratio_. Complex Networks panel: *N*: number of voxels in the aortic domain; *l*_*ij*_: Euclidean distance between voxels *i* and *j*; *N*_LC_: number of nodes of the network’s largest connected component, i.e., the maximal set of nodes such that each pair of nodes is connected by a path. An explanatory example of a network is shown, where node *1* is characterized by high hemodynamic degree centrality, the shortest path between nodes *3* and *4* is in green while the other links in blue.

Lumen segmentation of the thoracic aortas was performed on phase-contrast enhanced MR angiogram using ITK-Snap and used for a centerline-based reconstruction of 3D aortic geometries in VMTK 1 (www.vmtk.org). Anatomic landmarks were identified from co-registered 2D cine images and used to ensure a consistent spatial extent across all patients,^8^,^21^ defining the AAo, the aortic arch, and the descending aorta (DAo) portions used for the CNs analysis (Fig. 1). The reconstructed 3D geometries were used as masks for the acquired velocity data.^47^

### Quantitative Hemodynamic Descriptors

The acquired phase-velocity data were used to describe the spatiotemporal heterogeneity of large-scale dominant aortic flow features. The time-histories along the cardiac cycle of three intravascular hemodynamic quantities (Fig. 1) were derived from *in vivo* data: (1) the local velocity magnitude|**V**|; (2) the axial component *V*_ax_ of the local velocity vector **V** (or local “through-plane” velocity), defined as the projection of **V** along the direction of the tangent to the local vessel centerline^8^,^33^; (3) the secondary component *V*_sc_ of the local velocity vector (or local “in-plane” velocity), orthogonal to the local axial direction and related to secondary flows.^33^ A positive value of *V*_ax_ indicates forward flow (along the main, i.e., proximal-to-distal, flow direction), whereas a negative value is representative of retrograde flow.^8^ Similarly, positive and negative values of *V*_sc_ indicate in-plane right- and left-handed direction, respectively, when viewed in the direction of the forward movement.^33^

Geometric and hemodynamic quantities measurable in a clinical framework were also evaluated from 4D flow MRI data (Fig. 1) and will be referred to as “clinical parameters” from now on. In detail, the ratio between the maximum AAo diameter *D*_max_ (distal to the aortic root), a largely adopted clinical indicator of aortic dilation,^17^ and the aortic root maximum diameter *D*_root_ was measured and indicated with *D*_ratio_. Moreover, the acquired phase-velocity data were used to quantify the AAo systolic flow eccentricity expressed in terms of flow jet angle (FJA) and normalized flow displacement (FD), as defined elsewhere.^45^ Pulse wave velocity (PWV), considered the gold standard for measuring arterial stiffness,^17^ was non-invasively quantified from 4D flow MRI data in the AAo using a wavelet-based method described elsewhere.^2^,^23^ As a measure of the energy associated with the large-scale aortic flow features, cycle-average and peak blood flow kinetic energy (KE) were computed as:

1 $${\text{KE}}_{avg} = \frac{1}{V}\int\limits_{V} {\frac{1}{2}\rho \left| {\mathbf{V}} \right|_{avg}^{2} dV = \frac{1}{V}\int\limits_{V} {\frac{1}{2}\rho } } \left[ {\frac{1}{T}\mathop \smallint \limits_{0}^{T} \left| {{\mathbf{V}}\left( t \right)} \right|dt} \right]^{2} dV$$

and

2 $${\text{KE}}_{peak} = \frac{1}{V}\int\limits_{V} {\frac{1}{2}\rho \left| {\mathbf{V}} \right|_{peak}^{2} dV} ,$$

respectively. Both KE_avg_ and KE_peak_ quantities are averaged over the aortic volume *V*. In Eqs. (1) and (2), *ρ* is the blood density, $|{\mathbf{V}}|_{\text{avg}}$ is the cycle-average magnitude of the velocity vector **V**(*t*), *T* is the period of the cardiac cycle, and $\left| {\mathbf{V}} \right|_{\text{peak}}$ is the magnitude of the velocity vector at peak systole.

Finally, a centerline-based analysis was also carried out to obtain aortic mean curvature ($\overline{\kappa }$) and torsion $(\overline{\tau})$.^41^

### *In Vivo* CNs Analysis

The spatiotemporal heterogeneity of the aortic intravascular flow was investigated by using 4D flow MRI data to build patient-specific correlation-based CNs, according to the scheme recently applied to *in silico* data.^7^,^8^,^13^ For each patient, three CNs were built from |**V**|, *V*_ax_ and *V*_sc_ time-histories along the cardiac cycle. Each node of a CN was defined by the voxel belonging to the aortic fluid domain where the time-history of the considered hemodynamic quantity was acquired. Two nodes *i* and *j* were considered to be connected by a topological link {*i*, *j*} if the Pearson correlation coefficient R_*ij*_ between time-histories at those nodes was greater than a threshold value $\hat{R}$ (Fig. 1). In this study, the threshold values adopted for the construction of the three CNs were selected based on a dataset of computational hemodynamic models of healthy human aortas.^13^,^20^,^33^,^34^ In detail, the median values of the distributions of the correlation coefficients among simulated time-histories were adopted as threshold values for *in vivo* CNs construction, resulting in $\hat{R}$ = 0.87 for |**V|**, $\hat{R}$ = 0.82 for *V*_ax_, and $\hat{R}$ = 0.03 for *V*_sc_ (*V*_sc_ correlation coefficients symmetrically distributed around zero). Based on these thresholds, each correlation matrix was converted into an adjacency matrix according to the criterion:

3 $$ {\text{A}}_{ij} = \left\{ {\begin{array}{*{20}l} {0,\, if\,{\text{R}}_{ij} \le \hat{R}\,or\, i = j,   } \\ {1,\,if\,{\text{R}}_{ij} > \hat{R}.} \\ \end{array}   } \right. $$

Matrix A_*ij*_ contains the information on nodes connections: A_*ij*_ = 1 (i.e., nodes *i* and *j* are connected by a link) if ${\text{R}}_{ij}$ > $\hat{R}$, and A_*ij*_ = 0 elsewhere (Fig. 1). For each patient, CNs metrics were applied to characterize the topological structure of |**V|**, *V*_ax_ and *V*_sc_ networks (Fig. 1). The first metric, the *hemodynamic degree centrality hDC*_*i*_ is defined as:

4 $$hDC_{i} = \frac{1}{N}\mathop \sum \limits_{i = 1}^{N} {\text{A}}_{ij} ,$$

where *N* is the number of voxels in the aorta. The quantity *hDC*_*i*_ measures the degree of homogeneity/heterogeneity of the velocity time-histories acquired at each voxel belonging to the CN with respect to the whole investigated fluid domain. Technically, it represents the number of nodes of the CN connected to node *i* (the so-called *nearest neighbors* of *i*), expressed as the percentage of the *N* voxels of the considered domain. Voxels where *hDC*_*i*_= 0 were not considered as CNs nodes because they had no connections with the rest of the network.

The other two CNs metrics provide a quantitative measure of the length of persistence of the correlation between velocity data time-histories.^8^,^13^ The *hemodynamic normalized average Euclidean distance hAED*_*i*_ (Fig. 1) is defined as:

5 $$hAED_{i} = \frac{1}{D} \frac{{\mathop \sum \nolimits_{j = 1}^{N} {\text{A}}_{ij} l_{ij} }}{{\mathop \sum \nolimits_{j = 1}^{N} {\text{A}}_{ij} }},$$

where *l*_*ij*_ is the Euclidean distance between node *i* and its neighbor *j*. To account for aortic geometric variability, *hAED*_*i*_ was normalized with respect to a reference diameter *D* (Table S1 of the Supplementary Data) identified for each patient as the AAo diameter of a representative healthy subject with the same age, gender and BSA.^12^ High *hAED*_*i*_ values indicate that the correlation between the phase velocity-based time-history at node (voxel) *i* and time-histories at its nearest neighbors persists for a large anatomical distance, while low *hAED*_*i*_ values indicate that all the connections of node *i* are confined to nodes in a small neighborhood. The *shortest path length* d_*ij*_, i.e. the minimum number of links separating two generic nodes *i* and *j* of the network, was used to calculate the topological distance metric *average shortest path length ASPL* of the network^8^,^37^ (Fig. 1), defined as:

6 $$ASPL = \frac{1}{{N_{LC} \left( {N_{LC} - 1} \right)}}\mathop \sum \limits_{{j \in N_{LC} ,i \ne j}}^{{}} {\text{d}}_{ij} ,$$

where *N*_LC_ is the number of nodes of the network’s largest connected component, i.e., the maximal set of nodes such that each pair of nodes is connected by a path. Based on Eq. (6), *ASPL* is the average length of the shortest paths (i.e., sequence of consecutive links) in the largest connected network component. High *ASPL* values indicate that the connections inside the CN are sparse and that the correlation between the phase velocity-based time-histories persists for a short topological distance.

Linear regressions were used to identify relationships between clinical parameters and CN metrics. The quality of the regression was evaluated with the Pearson’s correlation coefficient R. Significance was assumed for *p* < 0.05.

## Results

### *In Vivo* Hemodynamic Analysis

**Figure 2:**
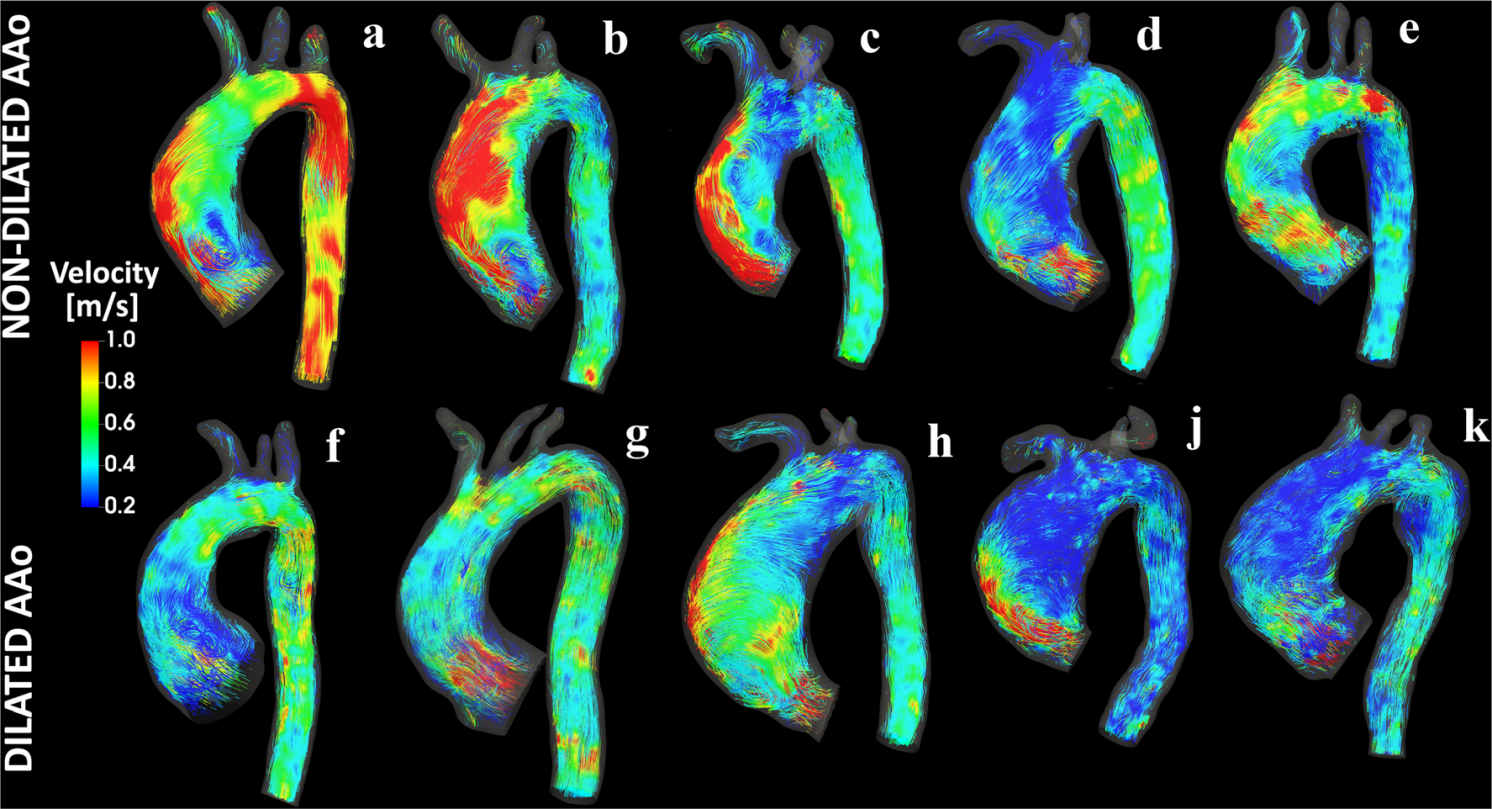
Visualizations of instantaneous streamlines at peak systole in patients with (bottom row) and without (top row) ascending aortic dilation. Colors represent velocity magnitude.

An overview of the large-scale aortic flow patterns in the ten patients object of this study is presented in Fig. 2 by means of instantaneous streamlines at peak systole. Streamlines visualization highlights a more intricate hemodynamics in dilated patients. Patients with BAV (A and H), those affected by valvular dysfunctions (B, C, D, E and K), and dilated AAo patient J exhibit an eccentric valvular outflow jet impinging on the outer curvature of the AAo (Fig. 2).

The patient-specific analysis of the correlation coefficients ${\text{R}}_{ij}^{{\left| {\mathbf{V}} \right|}}$, ${\text{R}}_{ij}^{ax}$ and ${\text{R}}_{ij}^{sc}$ between all pairs of the three velocity-based hemodynamic quantities |**V**|, *V*_ax_ and *V*_sc_ time-histories, respectively, is presented in Figure S1 of the Supplementary Data. The strongest correlations are registered between |**V**| time-histories, whereas *V*_ax_ correlation coefficients present lower median values due to the presence of anti-correlated waveforms (i.e., negative ${\text{R}}_{ij}^{ax}$ values). The correlation between *V*_sc_ time-histories is symmetrically distributed around the median, which is close to zero for all the patients. Overall, no marked differences can be appreciated between AAo non-dilated and dilated patients (Figure S1 of the Supplementary Data).

### *In Vivo* Complex Networks Analysis

**Figure 3:**
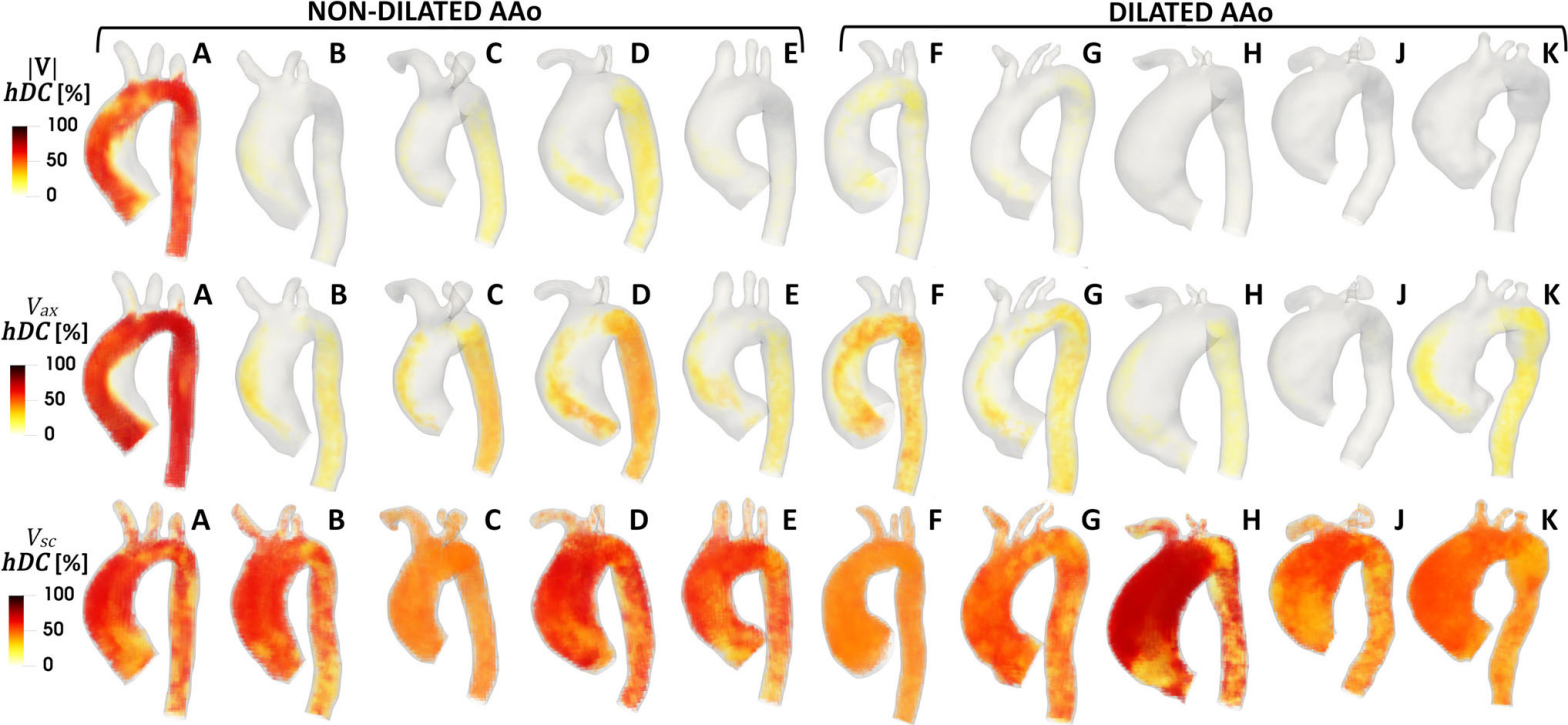
Volumetric maps of *hDC* for |**V**| (top row), *V*_ax_ (middle row) and *V*_sc_ (bottom row) CNs.

Volumetric maps of *hDC* (Fig. 3) highlight the spatiotemporal heterogeneity of the aortic blood flow. Most patients present with scarcely connected networks (low *hDC*), reflecting from scarce (as in dilated patients H, J and K, Fig. 3) to moderate homogeneity of |**V**| and *V*_ax_ time-histories. On the other hand, in patient A (Fig. 3) a dense pattern of connections between nodes (voxels) can be observed in the entire aortic domain (*hDC* values around 50%), indicating an overall high degree of similarity of velocity-based waveforms. In general, the aortic fluid domain is more connected in terms of *V*_ax_ than |**V**| and two dynamically-distinct regions in terms of *V*_ax_ waveforms can be identified: the first one in the DAo, where *V*_ax_ waveforms present moderate *hDC* values, and the second one at the AAo inner wall and aortic arch, where *V*_ax_ waveforms present low *hDC* values. The topologically isolated flow structure identified at the AAo inner wall by close-to-zero *hDC* values (Fig. 3) highlights the capability of the CNs approach in capturing large-scale blood flow disturbances: at the AAo inner wall the shape of *V*_ax_ time-histories along the cardiac cycle is markedly different from the overall aortic hemodynamics. This is the consequence of the combined effect of AAo curvature and aortic valve flow eccentricity, concurring to generate flow separation and recirculation at the inner wall, which in turn interact at the interface with the valve jet shear layer.

Denser patterns of connection between voxels characterize *V*_sc_ with respect to |**V**| and *V*_ax_ time-histories, indicating that *V*_sc_ CNs are more spatiotemporally compact than |**V**| and *V*_ax_ CNs and are characterized by the absence of topologically isolated regions (*hDC* higher than 40% in general, Fig. 3). No marked differences emerged between dilated and non-dilated patients (Fig. 3).

**Figure 4:**
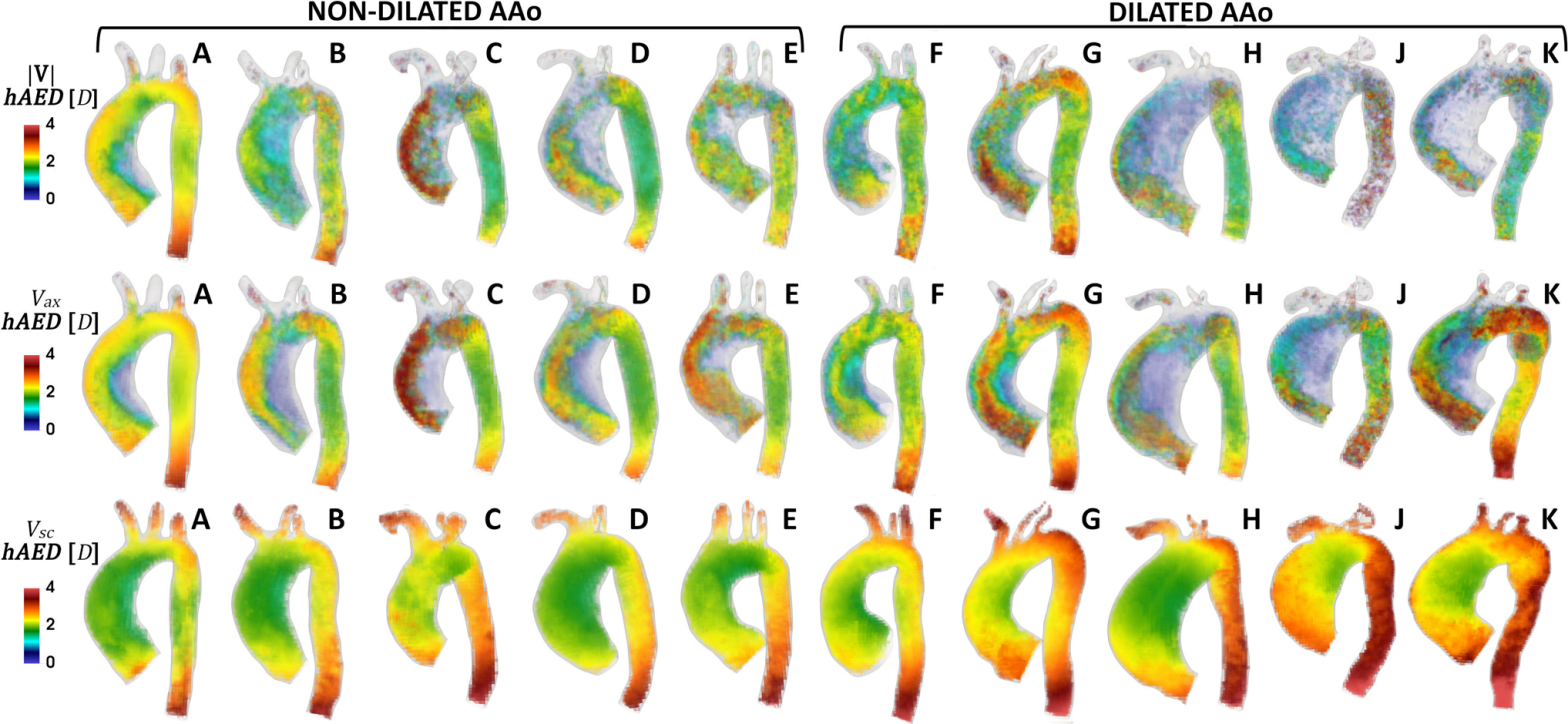
Volumetric maps of *hAED* for |**V**| (top row), *V*_ax_ (middle row) and *V*_sc_ (bottom row) CNs.

By visual inspection of the volumetric maps of *hAED*, quantifying the length of persistence of the correlation in the fluid domain (Fig. 4), in all patients |**V**| and *V*_ax_ time-histories located close to the AAo inner wall, where typically flow reversal occurs, are characterized by low *hAED* values. In the outer region of the AAo and in the Dao, |**V**| and *V*_ax_ networks are characterized by a neighborhood expanding on longer anatomical distances, confirming the persistence of more correlated flow structures in those regions (Fig. 4).

**Figure 5:**
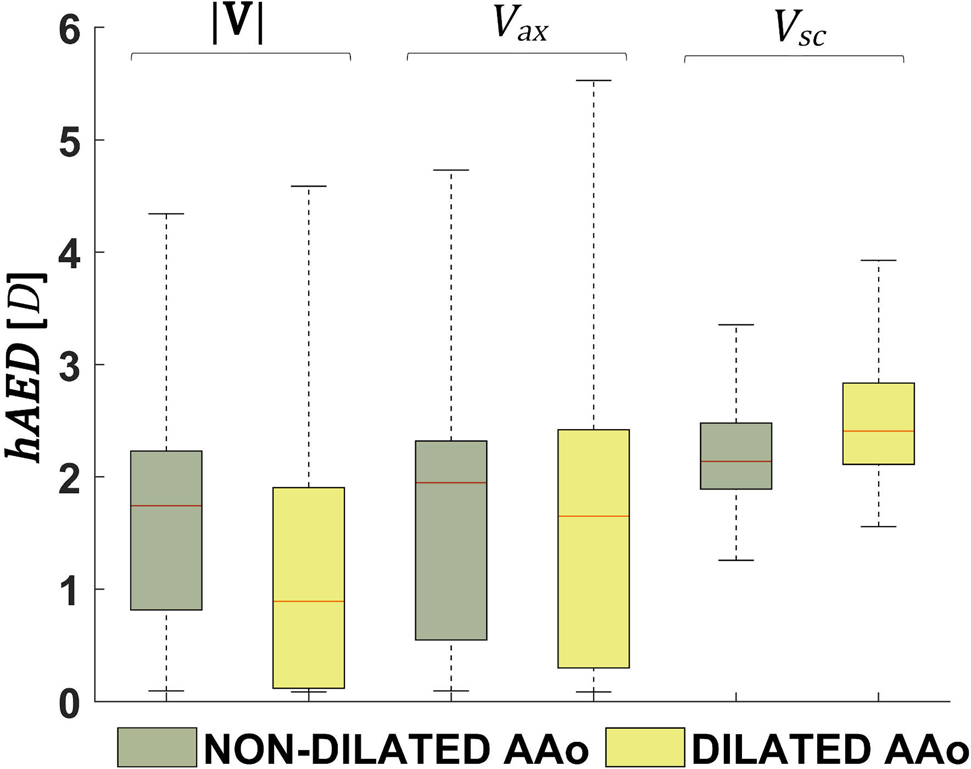
Effect of AAo dilation on the distributions of *hAED* values for |**V**|, *V*_ax_ and *V*_sc_ CNs. The median is indicated by the red line, the box indicates the interquartile range and the whiskers indicate the extreme values of the distribution.

Figure 5 quantitatively confirms the observation that in general the anatomical length of correlation persistence: (1) is longer in *V*_ax_ networks than in |**V**| networks; (2) in *V*_ax_ and |**V**| networks it is shorter in dilated compared to non-dilated patients; (3) in both *V*_ax_ and |**V**| networks it is shorter than two reference diameters *D* (|**V**| median values = 1.74 *D* and 0.89 $D$, and *V*_ax_ median values = 1.95 *D* and 1.65 *D*, for non-dilated and dilated patients, respectively). On the opposite, both dilated and non-dilated *V*_sc_ networks are characterized by an anatomical length of correlation persistence longer than two reference diameters *D* (median values = 2.13 *D* and 2.41 *D*, for non-dilated and dilated patients, respectively). Moreover, non-dilated patients present with median *hAED* shorter than dilated ones (Fig. 5), suggesting a major contribution for AAo dilation in shaping secondary blood flow patterns.

The analysis of the topological separation of the largest connected fluid structures in the aortic fluid domain, as quantified by the metric *ASPL* (Table 2), confirms the findings of the anatomy-based investigation summarized in Figs. 4 and 5. In detail, it emerges that in general two generic voxels of the largest component of the |**V**| networks are separated by a median of 3.05-links path in non-dilated patients, and by a 4.83-links path in dilated patients (Table 2), suggesting a role for AAo dilation in breaking up topological links. Although with a less marked difference, median *ASPL* values in the largest connected components of the *V*_ax_ networks reflect the results of the |**V**| networks (3.73- and 3.63-links path, in dilated and non-dilated patients, respectively, Table 2). Overall, the topological separation of the *V*_sc_ networks is not sensitive to AAo dilation (1.53- and 1.51-links path, in dilated and non-dilated patients, respectively, Table 2).

**Table 2:** *ASPL* values for |**V**|, *V*_ax_ and *V*_sc_ CNs.

	Patient	|V| *ASPL*	*V*_ax_ *ASPL*	*V*_sc_ *ASPL*
Non-dilated AAo	A	1.94	1.97	1.49
	B	3.05	3.96	1.51
	C	3.01	3.63	1.54
	D	3.41	2.83	1.47
	E	3.33	3.66	1.51
	Median	3.05	3.63	1.51
Dilated AAo	F	2.97	2.97	1.55
	G	3.30	3.03	1.53
	H	4.83	4.76	1.42
	J	5.78	5.17	1.53
	K	5.75	3.73	1.54
	Median	4.83	3.73	1.53

### Relationships Between Complex Networks Metrics and Clinical Parameters

**Figure 6:**
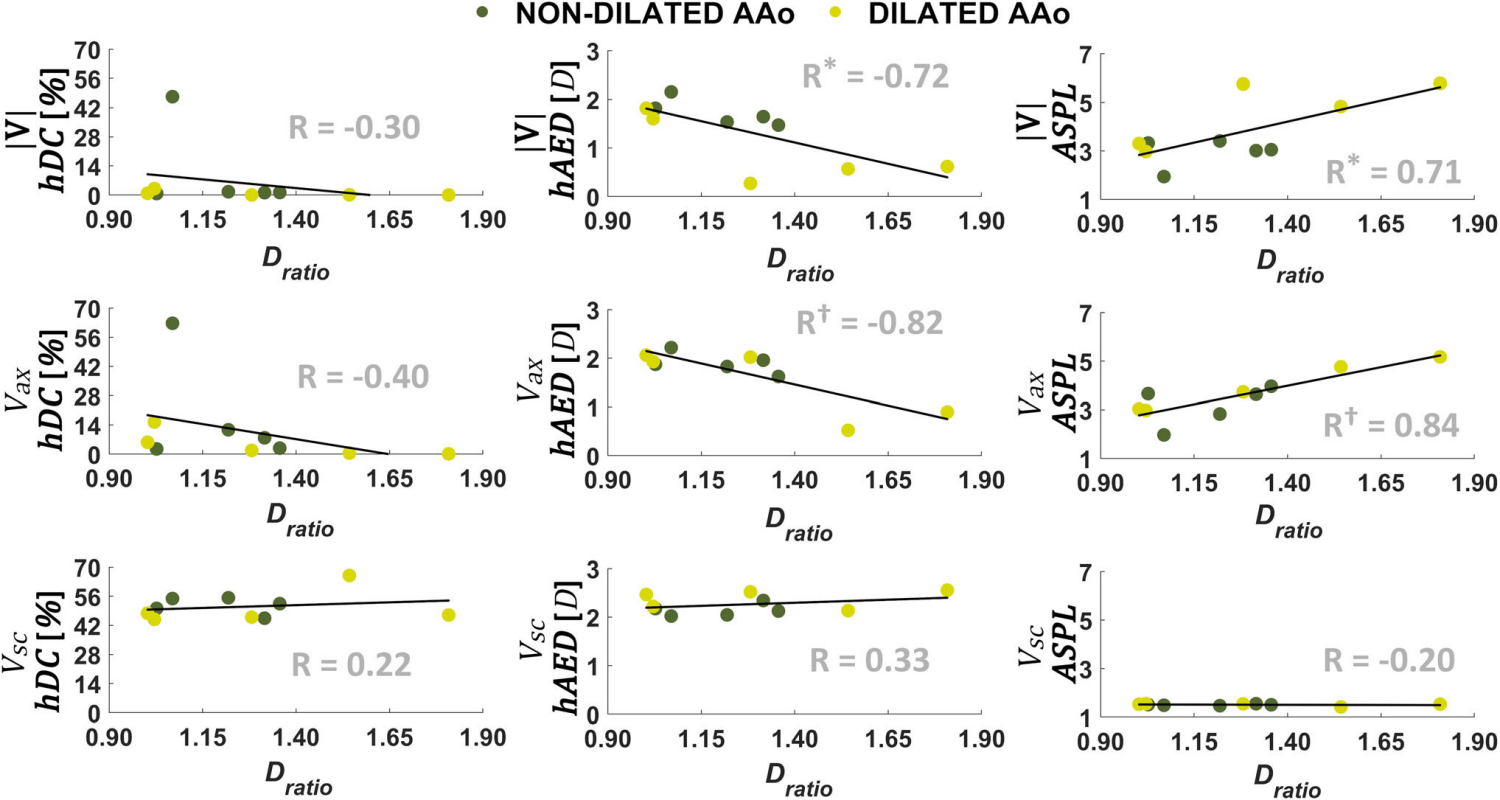
Associations between the geometric clinical indicator *D*_ratio_ and CNs metrics *hDC* (left column), *hAED* (middle column), and *ASPL* (right column) for |**V**| (top row), *V*_ax_ (middle row) and *V*_sc_ (bottom row) CNs. For each CN, *hDC* and *hAED* are expressed as the median value of all voxels. R: Pearson’s correlation coefficient (*p < 0.05, ^†^*p *< 0.01).

Significant associations emerge between the geometric clinical parameter *D*_ratio_ and the CNs metrics. In detail, *D*_ratio_ is negatively associated with *hAED* median values characterizing both |**V**| and *V*_ax_ networks (*p *= 0.020 and *p *= 0.004, respectively), suggesting a role for *D*_ratio_ in disrupting the spatiotemporal hemodynamic similarity of |**V**| and *V*_ax_ waveforms (Fig. 6). The results obtained considering the anatomical distances in the networks are confirmed by the topological analysis: a positive association emerges between *D*_ratio_ and *ASPL* values characterizing both |**V**| and *V*_ax_ networks (*p *= 0.020 and *p *= 0.002, respectively), suggesting that the aortic dilation breaks the correlation between |**V**| and *V*_ax_ time-histories, increasing topological separation between the measured voxel-based velocity quantities.

No significant associations emerge between *D*_ratio_ and the *hDC* median values of the |**V**|, and *V*_ax_ networks (Fig. 6). No significant associations emerge between *D*_ratio_ and *V*_sc_ network metrics, a consequence of the low dispersion of the latter (median *hDC* range = [44.9%, 66.0%], *ASPL* range = [1.42, 1.55], median *hAED* range = [2.02 *D*, 2.56 *D*], Fig. 6).

**Figure 7:**
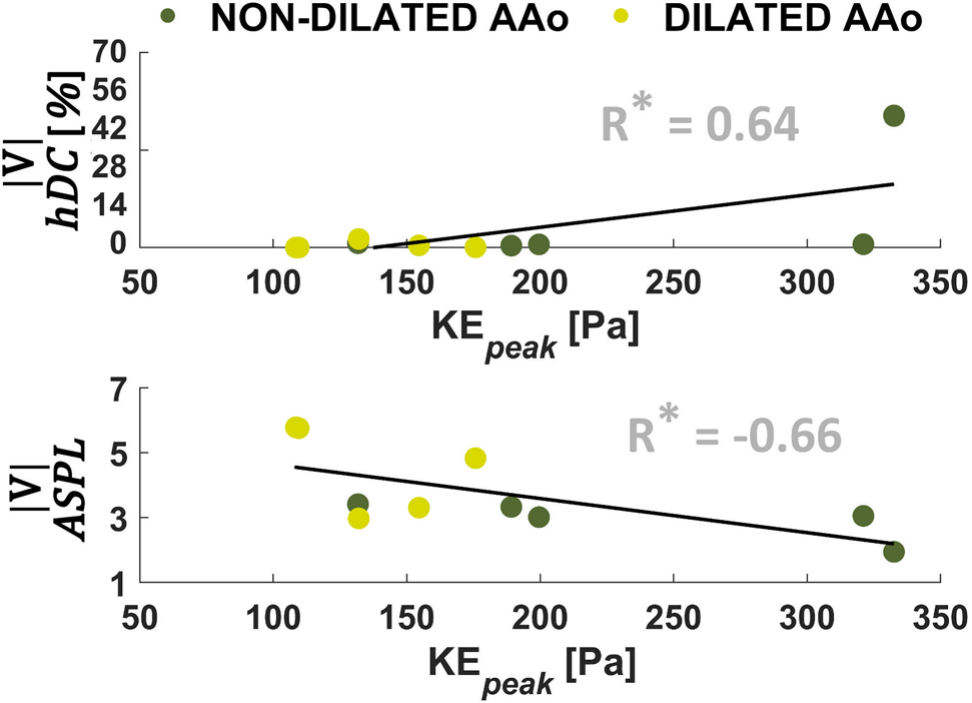
Associations of blood flow kinetic energy at the systolic peak (KE_peak_) with *hDC* (top) and ASPL (bottom) for |**V**| CNs. Here *hDC* is expressed as the median value of all voxels. R Pearson’s correlation coefficient (*p < 0.05, ^†^*p *< 0.01).

When considering the hemodynamic clinical parameters, a significant (albeit moderate) positive association emerges between blood flow KE at peak systole and *hDC* median values characterizing the |**V**| networks (*p *= 0.04), likely driven by the high *hDC* median value of patient A (rightmost point in Fig. 7, upper panel, resulting from the high *hDC* values in the whole domain as depicted in the *hDC* volumetric map of Fig. 3). A negative (albeit moderate) association emerges between KE at peak systole and *ASPL* values characterizing the |**V**| networks (*p *= 0.04, Fig. 7). Taken together, these results suggest that high KE_peak_ values might contribute to increase the spatiotemporal homogeneity and topological length of correlation persistence of the velocity vector field, in aorta. Neither the other investigated hemodynamic clinical parameters nor the centerline-based aortic geometric attributes $\overline{\kappa }$ and $\overline{\tau }$ impact the spatiotemporal heterogeneity of phase velocity data, as no significant correlations emerge with CNs metrics (Figures S2–S7 of the Supplementary Data).

## Discussion

The onset/progression of AAo wall dilation is the result of a complex multifactorial process in which local disturbed aortic hemodynamics plays a relevant role.^6^,^13^,^43^ In recent years, 4D flow MRI has been frequently employed for aortic flow visualization and/or characterization, aiming at elucidating the link between hemodynamic alterations and AAo disease *in vivo*.^14^,^25^,^43^

Stimulated by the need for interpretation of the intricate 4D aortic hemodynamics, and the identification of hemodynamic indicators/predictors of vascular disease to be adopted in a clinical framework, the current approach does not limit its focus on instantaneous snapshots or time- and space-average quantities, but takes into account the information embedded in the dynamical evolution of the aortic hemodynamics. To extend and deepen the *in vivo* characterization of hemodynamic complexity in the human aorta, the present study aims at investigating the spatiotemporal heterogeneity of large-scale aortic fluid structures by applying for the first time the Complex Networks theory to *in vivo* measured velocity data belonging to a 4D flow MRI dataset of human aortas with and without AAo dilation. In doing that, the CNs approach captures the large-scale “coherent flow structures” by implementing their definition as structures over which one fundamental macroscopic quantity (in this case, a velocity component or its magnitude) exhibits significant correlation with itself over a range of space and/or time significantly larger than the smallest scales of flow.^42^ The here-proposed CNs approach has the advantage of adding quantitative information to the detected coherent flow structures, represented by the anatomic and topological length of persistence of the correlation between the dynamical evolutions of the investigated quantities.

### CNs-Based Analysis of Aortic Flow Spatiotemporal Heterogeneity

**Figure 8:**
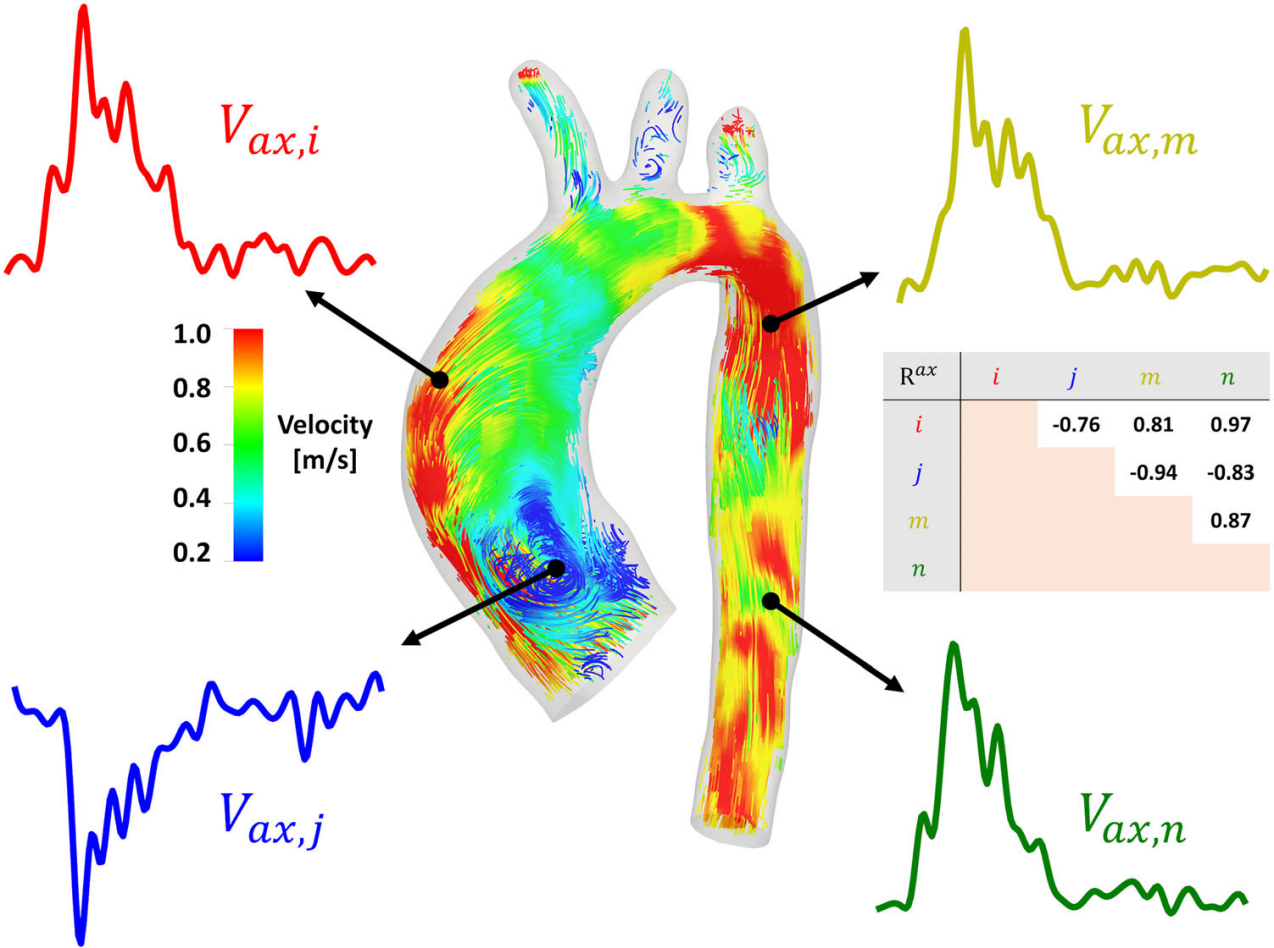
Explanatory example of differently correlated pairs of *V*_ax_ time-histories in a representative case. Regions of flow reversal, like the inner AAo, are characterized by *V*_ax_ waveforms with negative values (*V*_ax,*j*_ in the Figure), which are anti-correlated (negative R^ax^ values) with *V*_ax,*j*_ waveforms mainly aligned with the forward flow direction (*V*_ax,*i*_, *V*_ax,*m*_ and *V*_ax,*n*_ in the Figure). *V*_ax_ waveforms shown in the Figure were interpolated using a higher number of time points for visualization purposes.

Among the main findings, it is here reported that most of the patients (with the exception of non-dilated patient A) present with large spatiotemporal heterogeneity of both |**V**| and *V*_ax_ time-histories along the cardiac cycle, as the corresponding networks are characterized by very sparse connections and low degree of similarity (low *hDC*) between waveforms (Fig. 3). The distinguishable topologically isolated flow structures (*hDC* close to zero) identifiable by visual inspection at the AAo inner wall in the *V*_ax_ networks of all investigated patients (Fig. 3) can be explained by the presence of large-scale flow recirculation patterns.^19^,^31^,^46^ Such flow features have been associated to (1) intimal lipid accumulation^22^ in murine and rabbit thoracic aorta, and (2) atherosclerotic and thrombotic biological markers in an arterial replication platform.^32^ As explained in Fig. 8 for a representative case, the marked flow reversal is highlighted by *V*_ax_ waveforms with negative values, which are likely to be anti-correlated with *V*_ax_ waveforms mainly aligned with the forward flow direction, resulting in negative ${\text{R}}_{ij}^{ax}$ values (Figure S1). Interestingly, the symmetry of the *V*_sc_ correlation distribution around zero for all patients (Figure S1 of the Supplementary Data), taken together with the dense patterns of connections (higher *hDC*) in *V*_sc_ CNs, hints at the presence of a Dean-like secondary flow organization.^35^ These findings, while preliminary, suggest that the aortic secondary flow patterns are more homogeneous than velocity magnitude and axial flow.

### The Impact of AAo Dilation on the Persistence Length of Correlation in Velocity Data

The analysis of the spatial persistence of the correlation of aortic large-scale flow structures confirms the spatiotemporal heterogeneity of velocity data highlighted by *hDC* (Fig. 3). Of relevance, the anatomic length of persistence of the correlation *hAED* in *V*_sc_ networks is higher than |**V**| and *V*_ax_ networks (Figs. 4 and 5). In detail, the shape of *V*_sc_ time-histories presents with a high level of similarity at anatomical distances longer than 2*D*, a feature common both to dilated and non-dilated patients, while the similarity between |**V**| and *V*_ax_ time-histories is bounded below 2*D* (Fig. 5). The topological length of persistence of the correlation is also longer for *V*_sc_ than |**V**| and *V*_ax_ networks (as highlighted by *ASPL* values in Table 2).

The combined picture provided by the calculation of *hAED* and *ASPL* metrics suggests a different action for the AAo dilation on aortic velocity: a larger AAo dilation disrupts the (anatomical and topological) length of persistence of the similarity of both |**V**| and *V*_ax_ waveforms, while preserving more the spatiotemporal similarity of *V*_sc_ waveforms. These findings are in line with a recent study conducted on a dataset of computational hemodynamics models of healthy human carotid bifurcations,^8^ where a similar relation between the spatiotemporal heterogeneity of helical flow structures and a geometric indicator of carotid bulb expansion was reported.

The regression analysis conducted on CNs distance metrics and the geometric clinically measurable parameter *D*_ratio_ corroborates the hypothesis of an impact of AAo dilation on blood velocity. As for the *V*_sc_ networks, no significant association emerges between the aortic *D*_ratio_ and the network distance metrics *hAED* and *ASPL*. In other terms, the *V*_sc_ networks are more compact, less sensitive to AAo dilation than |**V**| and *V*_ax_ networks.

On the contrary, from the analysis of |**V**| and *V*_ax_ networks it emerges that the aortic *D*_ratio_ is negatively correlated with *hAED* (R = -0.72, *p *< 0.05 and R = − 0.82, *p *< 0.01, respectively, Fig. 6) and positively correlated with *ASPL* (R = 0.71, *p *< 0.05 and R = 0.84, *p *< 0.01, respectively, Fig. 6). From a fluid mechanics viewpoint, the present analysis demonstrates that AAo dilation, promoting the development of flow disturbances, has a detrimental effect on the spatiotemporal coherence of |**V**| and *V*_ax_ waveforms in the aorta. What emerges from the *in vivo* CNs-based quantitative analysis agrees with previous semi-quantitative studies reporting of an association (1) between age, AAo diameter and the presence of large vortices in aorta,^18^ and (2) between the presence and strength of vortical and helical flow patterns and AAo diameter and AAo/DAo diameter ratio.^6^ Interestingly, and consistently with the findings of this *in vivo* analysis, a very recent study integrating CNs with computational hemodynamics simulations suggests that the length of correlation persistence in forward flow could be shorter in the presence of AAo dilation, compared to the healthy ascending aorta.^13^ The emergent picture leads to presume that the physiological spatiotemporal coherence of large-scale forward flow could be progressively compromised in the presence of increasing AAo dilation. The capability of the CNs analysis to underscore intravascular flow spatiotemporal heterogeneity and disturbed flow features like flow separation and recirculation, increased velocity jets, highly rotational flow, allows a deeper understanding of the interaction of these intravascular fluid structures with the vascular wall, and ultimately near-wall biological transport and endothelial-cell mediated pathways involved in vascular remodeling and dilation.^30^,^33^,^35^,^40^,^49^ In addition to that, given the importance of intravascular flow features in terms of flow energetics, the correlation persistence, measured by CNs, as well as the amount of spatiotemporal heterogeneity in the flow field, is expected to have an impact in the ventricular work.

A main limitation that could potentially weaken the findings of this study is the choice of the correlation threshold used to build the CNs. Consistently with previous CNs-based in silico studies,^7^,^8^,^13^ the CNs threshold for a specific hemodynamic quantity was set equal to the median value of the correlation coefficients distribution obtained from a dataset of computational hemodynamic models of healthy aortas. Further studies on a 4D flow MRI dataset of healthy subjects could be useful to investigate the robustness of the selected thresholds. Although the noise typically affecting 4D flow MRI acquisitions could cause an underestimation of the correlations between velocity waveforms, the pattern of connection between nodes resulting from the threshold-based approach adopted in this study to build the CN is expected to be marginally influenced by the use of *in vivo* data as acquired. In addition to that, the systolic phase, which is expected to drive the correlations, is characterized by a higher signal-to-noise ratio than the diastolic phase, therefore dampening the impact of noise on the correlation distribution. Another limitation of this study lies in its cross-sectional nature, which does not allow to draw any conclusion about the potential of the approach as an *in vivo* tool for risk prediction, for which longitudinal studies are needed. However, this was intended as an exploratory study aiming at testing the ability of the CNs to characterize *in vivo* complex aortic flows and their link to vascular disease. All patients enrolled for this study presented with aortic valvular dysfunctions (from mild to severe), and two of them with BAV (Table 1), thus preventing a robust comparison between valve-mediated hemodynamics in BAV vs. TAV groups. Moreover, the scarce stratification as well as the small sample size could have polarized the regression analysis results involving *hDC* and influenced the associations between CNs metrics and the other investigated clinical hemodynamic parameters and centerline-based geometric attributes. In addition to that, based on the scatter plots in Figs. 6, 7 and in the Supplementary Data, non-dilated patient A appears as an outlier/leverage point in relation of the *hDC* metric for the |**V**| and *V*_ax_ networks. However, when removing patient A from the regression analysis, the observed statistical significance (Fig. 6) of the associations between CNs distance metrics and *D*_ratio_ is preserved, whereas the correlations with KE_peak_ in the |**V**| CNs (Fig. 7) become non statistically relevant. Therefore, further investigations with larger and better stratified datasets are needed to confirm the present findings on the spatiotemporal heterogeneity of large-scale flow features.

In conclusion, in this study the CNs theory was applied to *in vivo* velocity data from a 4D flow MRI dataset of human aortas to (1) investigate the spatiotemporal heterogeneity of large-scale aortic flow structures and (2) assess the existence of possible associations between CNs metrics and ascending aortic dilation. Results showed that velocity magnitude and through-plane (axial) velocity structures are characterized by a larger spatiotemporal heterogeneity than in-plane (secondary) flow structures. Moreover, an increasing AAo dilation disrupts the correlation in forward flow reducing the correlation persistence length, while preserving the spatiotemporal homogeneity of secondary flows. The here presented approach for obtaining *in vivo* measurable information on aortic hemodynamics by integrating 4D flow MRI and CNs in a clinical framework shows a strong potential as a tool for visualization and quantification of complex cardiovascular flows, and the use of CNs distance metrics may allow a finer risk stratification of AAo disease.

## Supplementary Information

Below is the link to the electronic supplementary material.

Supplementary file 1 (PDF 866 kb).
