# Supplementary material for: Combining 4D Flow MRI and Complex Networks Theory to Characterize the Hemodynamic Heterogeneity in Dilated and Non-dilated Human Ascending Aortas
Source: Ann Biomed Eng. 2021 Jun 2;49(9):2441–53. doi: 10.1007/s10439-021-02798-9 (PMC8455395; doi:10.1007/s10439-021-02798-9)
Supplement: Supplementary file 1 — (PDF 866 kb). [file 10439_2021_2798_MOESM1_ESM.pdf]

Supplemental Materials (Not to be Published)

Supplementary Data

- **Supplementary Data** -

Combining 4D Flow MRI and Complex Networks Theory to Characterize the Hemodynamic Heterogeneity in Dilated and Non-Dilated Human Aortas

Karol Calò[1], Diego Gallo[1], Andrea Guala[2,3], Jose Rodriguez Palomares[2,3,4], Stefania Scarsoglio[1], Luca Ridolfi[1], Umberto Morbiducci*[1]

[1] Polito[BIO]Med Lab, Department of Mechanical and Aerospace Engineering, Politecnico di Torino, Turin, Italy

[2] Vall d'Hebron Institut de Recerca (VHIR), Barcelona, Spain

[3] CIBER-CV, Instituto de Salud Carlos III (ISCIII), Madrid, Spain

[4] Department of Cardiology, Hospital Universitari Vall d'Hebron, Barcelona, Spain

***Address for correspondence:**

Umberto Morbiducci, Full Professor

Department of Mechanical and Aerospace Engineering, Politecnico di Torino

Corso Duca degli Abruzzi, 24

10129 Turin, Italy

Tel.: +39 011 0906882

Fax: +39 011 5646999

E-mail: umberto.morbiducci@polito.it

Supplementary Data

Supplementary Results

The patient-specific analysis of the correlation coefficients $\mathrm{R}_{ij}^{|\mathbf{V}|}$, R_{ij}^{ax} and R_{ij}^{sc} between all pairs of the three velocity-based hemodynamic quantities $|\mathbf{V}|$, V_{ax} and V_{sc} time-histories, respectively, is presented in Figure S1. The strongest correlations emerge between $|\mathbf{V}|$ time-histories, a feature common to all the patients (median values range = [0.27, 0.83]). Although three of five AAo dilated patients (H, J and K) present with the lowest $\mathrm{R}_{ij}^{|\mathbf{V}|}$ median values, no marked differences can be appreciated between AAo non-dilated and dilated patients. All patients present with median values of V_{ax} correlation coefficients lower than $|\mathbf{V}|$ time-histories (with the exception of patient K) and with larger interquartile range (Figure S1). This can be ascribed to the more marked presence of negative correlations between V_{ax} time-histories, compared to $|\mathbf{V}|$ time histories (Figure S1). The correlation between V_{sc} time-histories is symmetrically distributed around the median, which is close to zero for all the

patients (median values range = [0.00, 0.10]). This symmetry around zero in the distribution of the correlation coefficients between V_{sc} time-histories reflects the presence of overall balanced right- and left-handed secondary flow structures, characterized by V_{sc} values of opposite sign. From Figure S1 no remarkable differences emerge between AAo non-dilated and dilated patients' R_{ij}^{ax} and R_{ij}^{sc} distributions.

Supplementary Data

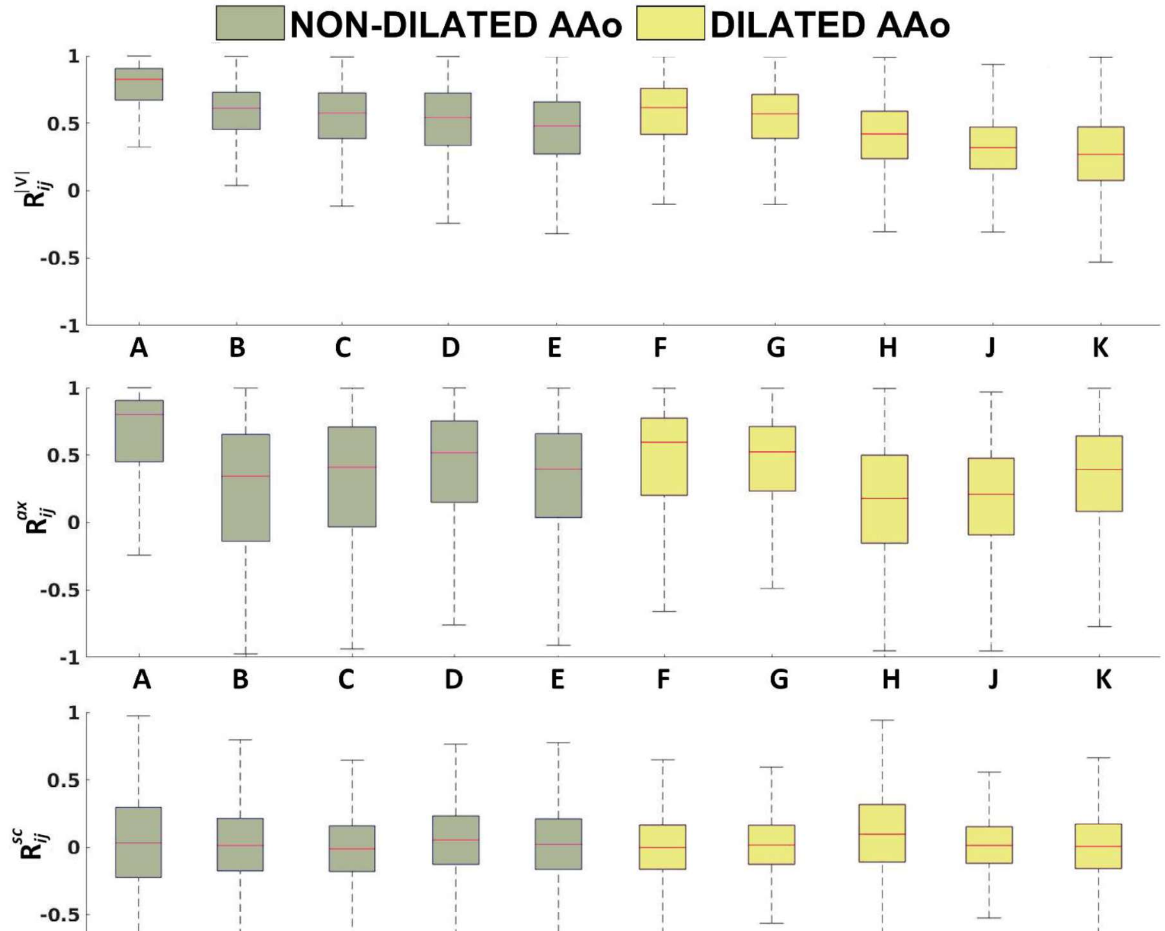

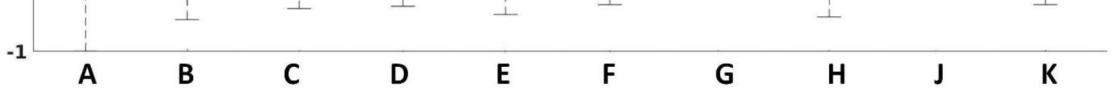

Figure S1. Distributions of the Pearson correlation coefficients between phase velocity time-histories in each model, for |**V**| (top row), V_{ax} (middle row) and V_{sc} (bottom row). The median is indicated by the red line, the box indicates the interquartile range and the whiskers indicate the extreme values of the distribution.

Supplementary Data

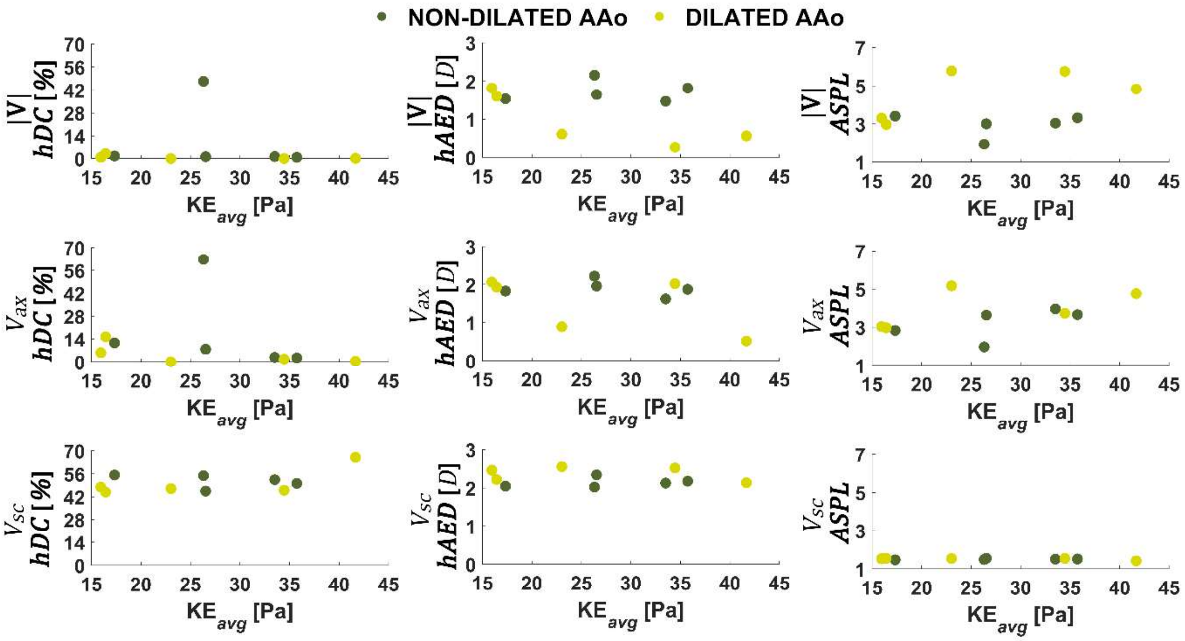

Figure S2. Associations between the cycle-average blood flow kinetic energy (KE_{avg}) and CNs metrics hDC (left column), $hAED$ (middle column), and $ASPL$ (right column) for $|\mathbf{V}|$ (top row), V_{ax} (middle row) and V_{sc} (bottom row) CNs. For each CN, hDC and $hAED$ are expressed as the median value of all voxels.

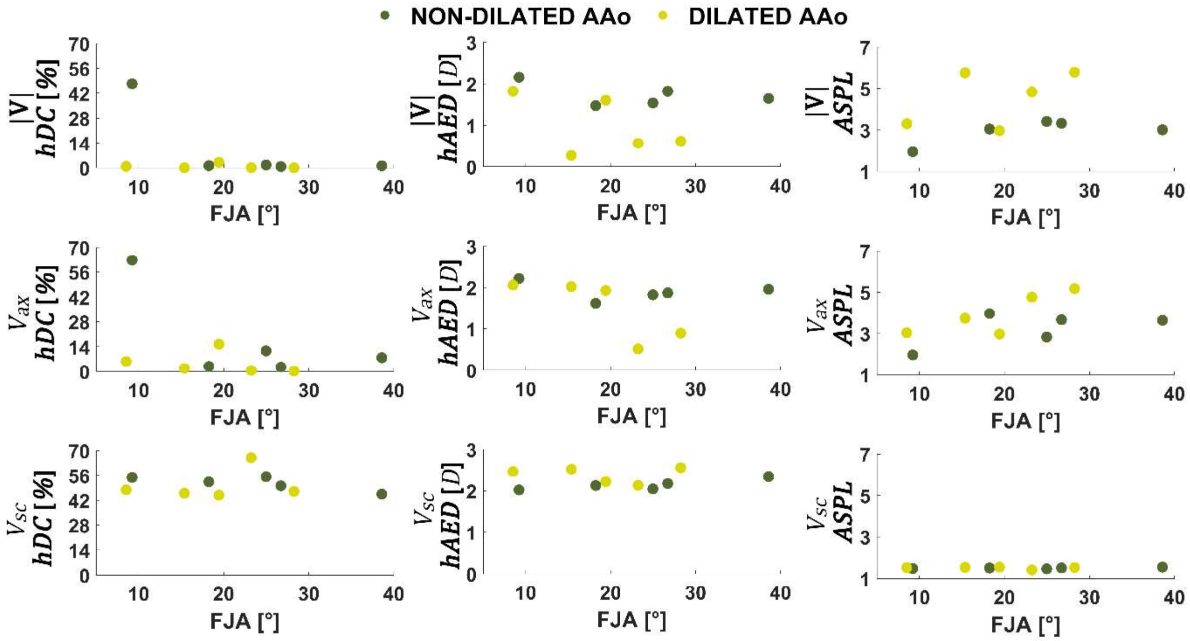

Figure S3. Associations between flow jet angle (FJA) and CNs metrics hDC (left column), $hAED$ (middle column), and $ASPL$ (right column) for $|\mathbf{V}|$ (top row), V_{ax} (middle row) and V_{sc} (bottom row) CNs. For each CN, hDC and $hAED$ are expressed as the median value of all voxels.

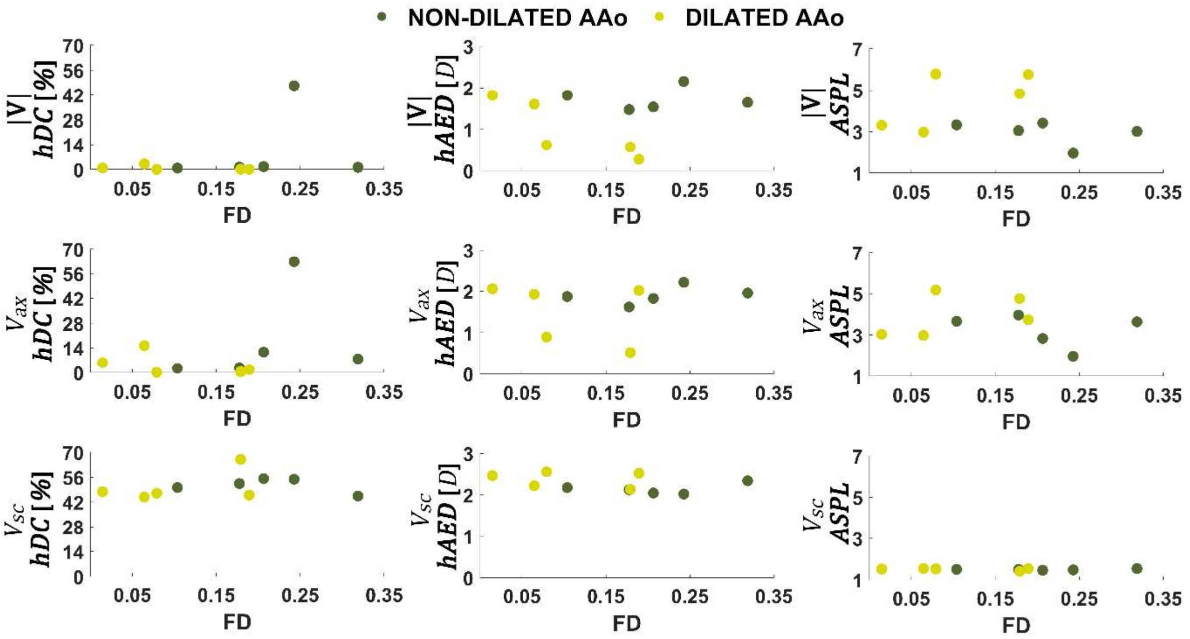

Figure S4. Associations between normalized flow displacement (FD) and CNs metrics hDC (left column), $hAED$ (middle column), and $ASPL$ (right column) for $|\mathbf{V}|$ (top row), V_{ax} (middle row) and V_{sc} (bottom row) CNs. For each CN, hDC and $hAED$ are expressed as the median value of all voxels.

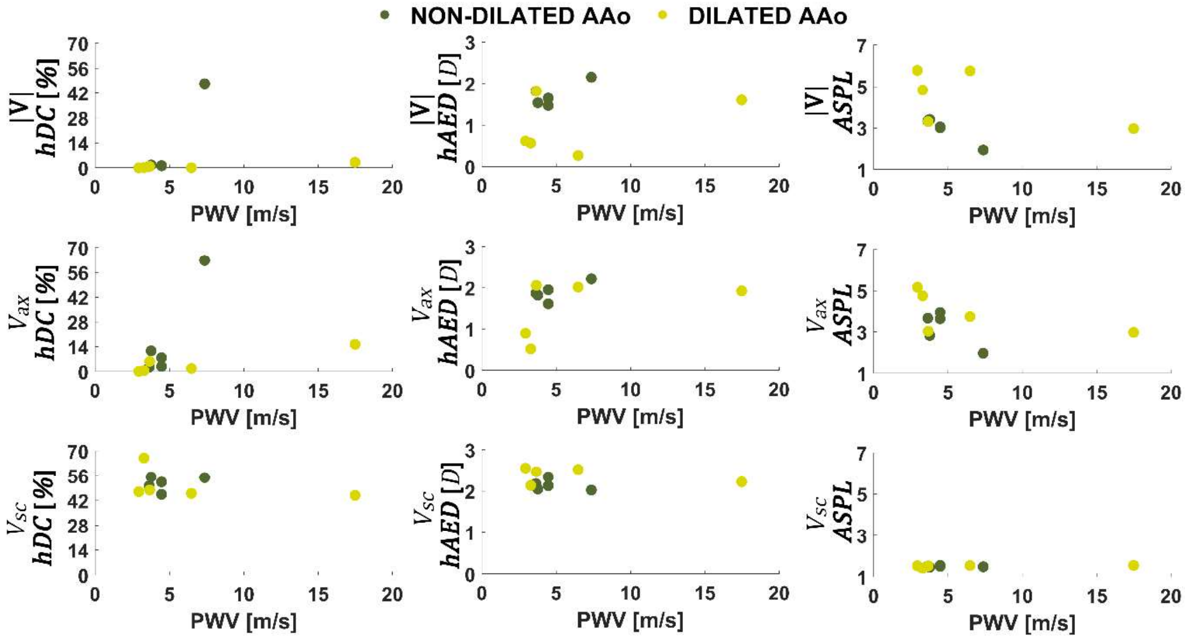

Figure S5. Associations between pulse wave velocity (PWV) and CNs metrics hDC (left column), $hAED$ (middle column), and $ASPL$ (right column) for $|\mathbf{V}|$ (top row), V_{ax} (middle row) and V_{sc} (bottom row) CNs. For each CN, hDC and $hAED$ are expressed as the median value of all voxels.

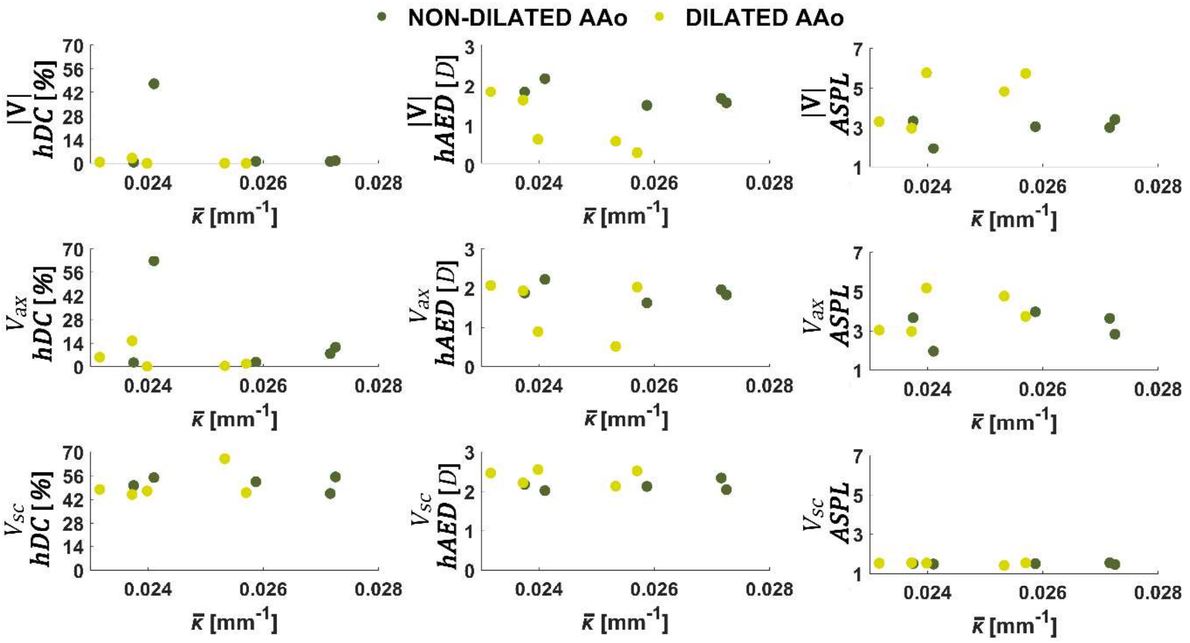

Figure S6. Associations between aortic mean curvature ($\bar{\kappa}$) and CNs metrics hDC (left column), $hAED$ (middle column), and $ASPL$ (right column) for $|\mathbf{V}|$ (top row), V_{ax} (middle row) and V_{sc} (bottom row) CNs. For each CN, hDC and $hAED$ are expressed as the median value of all voxels.

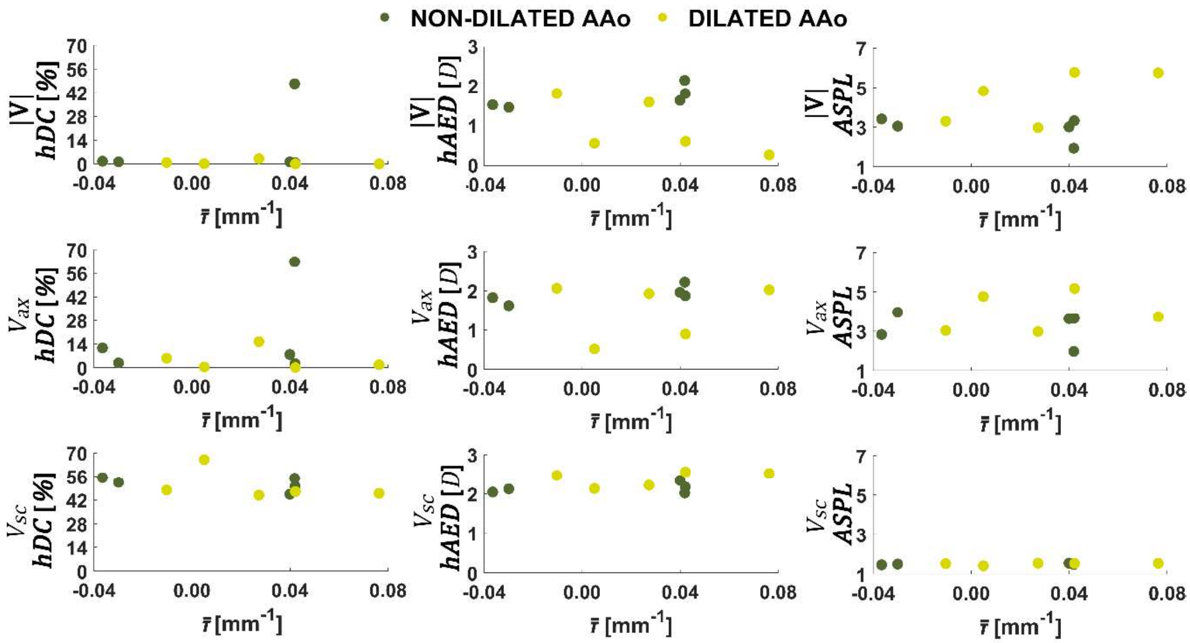

Figure S7. Associations between aortic mean torsion ($\bar{\tau}$) and CNs metrics hDC (left column), $hAED$ (middle column), and $ASPL$ (right column) for $|\mathbf{V}|$ (top row), V_{ax} (middle row) and V_{sc} (bottom row) CNs. For each CN, hDC and $hAED$ are expressed as the median value of all voxels.

Supplementary Data

Table S1

Demographics of the study population and AAo reference diameters D used for the definition of $hAED$.

**Patient**	**Gender**	**Age (years)**	**BSA (m^2)**	***Reference AAo diameter D (cm)**
**A**	Male	35	2.02	26.0
**B**	Female	82	-	24.7
**C**	Female	79	1.66	24.7
**D**	Female	55	-	24.7
**E**	Male	73	-	26.0
**F**	Male	41	2.31	28.5
**G**	Male	49	1.98	26.0
**H**	Male	65	1.79	26.0
**J**	Male	77	1.85	26.0
**K**	Male	67	2.08	27.4

* Based on a gender specific normal database of reference diameters for the aorta, from Davis et al.[1]

Supplementary References

1. Davis, A. E., A. J. Lewandowski, C. J. Holloway, N. A. B. Ntusi, R. Banerjee, R. Nethononda, A. Pitcher, J. M. Francis, S. G. Myerson, P. Leeson, T. Donovan, S. Neubauer, and O. J. Rider. Observational study of regional aortic size referenced to body size: production of a cardiovascular magnetic resonance nomogram. *J. Cardiovasc. Magn. Reson. Off. J. Soc. Cardiovasc. Magn. Reson.* 16:9, 2014.
